# The Effectiveness of Cerium Oxide Nanoparticle-Based Drugs in Wound Healing in Animal Models

**DOI:** 10.3390/molecules30234536

**Published:** 2025-11-24

**Authors:** Anna G. Erokhina, Maria P. Kruglova, Victor A. Stupin, Anton V. Tsaregorodtsev, Vladimir A. Parfenov, Natalia E. Manturova, Ekaterina V. Silina

**Affiliations:** 1 I.M. Sechenov First Moscow State Medical University (Sechenov University), 119991 Moscow, Russia; anna.8545@mail.ru (A.G.E.); kruglova_m_p@staff.sechenov.ru (M.P.K.); vladimirparfenov@mail.ru (V.A.P.); 2 Pirogov Russian National Research Medical University, 117997 Moscow, Russia; stvictor@bk.ru (V.A.S.); manturovanatali@yandex.ru (N.E.M.); 3 Bakulev Scientific Center for Cardiovascular Surgery, 121552 Moscow, Russia; avtsaregorodtsev@bakulev.ru

**Keywords:** rare earth metals, nanoparticles, nanocerium, cerium oxide, regeneration, antioxidant effect, proliferative effect, wounds, burns

## Abstract

Cutaneous regeneration remains a major challenge in biomedicine, prompting the exploration of novel therapeutic agents such as cerium oxide nanoparticles (CeO_2_ NPs, nanoceria). These nanoparticles exhibit multifaceted regenerative properties, including stimulation of metabolic and proliferative activity in keratinocytes, fibroblasts, and endothelial cells, potent antioxidant effects, immunomodulatory potential, and antimicrobial activity. Although numerous in vitro studies have characterized these properties, there is a critical need to evaluate nanoceria in more physiologically relevant in vivo settings, where dynamic biological conditions may significantly influence their efficacy. Furthermore, the therapeutic performance of CeO_2_ NPs is highly dependent on the synthesis methods and formulation components (excipients and co-administered active substances). A review of existing in vivo studies investigating nanoceria-based formulations for wound healing addresses this gap. The authors found 25 relevant studies published as of September 2025 in major scientific databases, including PubMed, Scopus, the Cochrane Library, which provided data on the effectiveness of using cerium oxide nanoparticles as components of medical devices or wound dressings in accelerating wound healing in animal models. This analysis synthesizes evidence on nanoparticle efficacy, formulation strategies, and observed biological outcomes across animal models. These findings indicate that nanoceria formulations can accelerate wound closure and modulate the key phases of tissue repair, although the outcomes vary with particle characteristics and delivery systems. While nanoceria hold considerable promise for clinical wound management, standardized reporting of synthesis protocols and rigorous comparative in vivo studies are essential to translate their potential into reliable therapeutic applications.

## 1. Introduction

Wound healing is a complex and dynamic process that requires the coordinated interplay of multiple phases, including hemostasis, inflammation, cellular maturation, migration and proliferation, angiogenesis, and remodeling of newly formed tissue [1]. Disruption at any stage (hemostasis, inflammation, proliferation, or remodeling) can lead to excessive connective tissue deposition and hypertrophic scarring or to chronic, non-healing wound development [2,3]. Although the understanding of wound regeneration mechanisms and therapeutic strategies continues to evolve and expand, impaired wound healing remains one of the most pressing challenges in modern medicine and healthcare. The trajectory and outcome of healing are influenced by numerous factors: wound type (e.g., surgical incision, burn, or traumatic injury) and severity of tissue damage [4,5,6]; comorbid conditions such as metabolic disorders or vascular disease [7,8,9]; immune status [10]; microbial contamination [11,12]; and the timeliness and appropriateness of clinical interventions [13]. Compounding this challenge, antimicrobial resistance (AMR) has emerged as a critical global health threat, significantly diminishing the efficacy of conventional antibiotics [14,15,16]. This growing resistance not only impedes tissue regeneration but can also worsen tissue damage, facilitate the spread of infection, and lead to limb amputation or life-threatening sepsis in severe cases [17,18]. Consequently, there is an urgent need to deepen our understanding of the biological mechanisms underlying wound repair and to develop innovative therapeutic approaches that address both impaired healing and AMR.

The use of rare-earth metal nanoparticles, particularly cerium dioxide (CeO_2_), is a promising strategy for addressing the dual challenges of impaired tissue regeneration and AMR. This substance is among the most extensively studied and biocompatible nanomaterials, exhibiting a range of beneficial properties, including regenerative, antibacterial, and antioxidant effects [19,20,21,22,23]. However, the therapeutic efficacy of CeO_2_ nanoparticles can vary significantly depending on their physicochemical characteristics, such as size, shape, surface charge, and crystallinity [24], as well as on the choice of excipients and auxiliary components used in the formulation of the final drug product, all of which may directly modulate the biological activity of nanoceria [25]. Moreover, the inflammatory response evolves dynamically throughout the healing process, with marked shifts in local conditions (cytokine profiles, pH, membrane surface charge, presence or absence of microbial contamination, and the relative dominance of exudative versus proliferative phases). These factors can profoundly influence the nanoparticle behavior and bioactivity. Therefore, the evaluation of the performance of CeO_2_ nanoparticles and their formulations must consider this biological complexity. Crucially, such assessments should be conducted under in vivo conditions that reflect the specific wound type and its pathophysiological context, as in vitro models alone cannot capture the dynamic interplay of factors governing healing in living systems.

Studies evaluating the regenerative potential of cerium dioxide nanoparticles in in vivo animal models are of particular value; however, such data remain scarce in the current literature. To address this gap, a review was conducted, which identified 25 relevant studies published up to September 2025 from major scientific databases, including PubMed, Scopus, Cochrane Library. The primary objective of this review was to analyze the existing evidence on the efficacy of cerium oxide nanoparticles, formulated as components of medical products or wound dressings, in promoting wound healing in animal models.

## 2. Results

### 2.1. Mechanisms Underlying the Wound-Healing Effects of Cerium Dioxide Nanoparticles

A growing body of evidence indicates that cerium dioxide nanoparticles exert their wound-healing effects through multiple interrelated mechanisms, including antioxidant activity, anti-inflammatory action, cellular proliferation and angiogenesis stimulation, and antibacterial properties. However, the magnitude and direction of these effects are highly context-dependent, and influenced by a range of physicochemical and biological variables.

**Figure 1:**
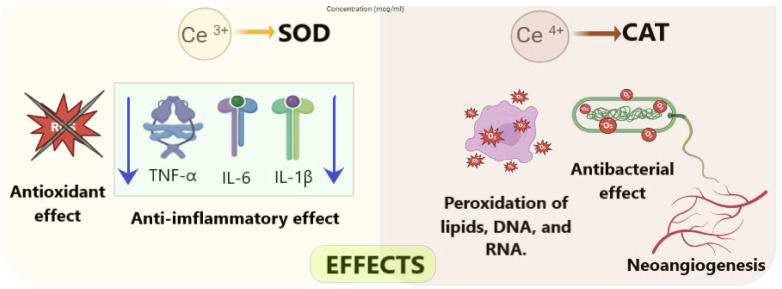
Nanoceria effects depending on its oxidation state. Created with BioRender.com (free for download).

The redox activity of cerium stems from its unique electronic structure and the presence of oxygen vacancies in its lattice determines its variable valence, which enable reversible switching between Ce^3+^ and Ce^4+^ oxidation states [26,27]. This redox flexibility underpins the dual enzymatic mimicry of the nanoceria. Depending on the predominant phase of cerium oxide, it can have different effects (Figure 1): Ce^3+^-rich surfaces exhibit superoxide dismutase (SOD)-like activity, converting superoxide radicals (O_2_•^−^) into hydrogen peroxide (H_2_O_2_), whereas Ce^4+^-dominant surfaces display catalase (CAT)-like activity, decomposing H_2_O_2_ into water and oxygen [28,29]. This tandem action enables nanoceria to mitigate reactive oxygen species (ROS)-induced cellular damage, a key factor in chronic non-healing wounds.

Nevertheless, the balance between Ce^3+^ and Ce^4+^ is not intrinsic but dynamically modulated by the local microenvironment, particularly pH. In living systems, considering the nature of the wound, the inflammatory response phase, and available microbial contamination is critical because these factors affect the pH of the wound environment and, consequently, the nanoparticle activity pattern. In chronic wounds characterized by alkaline conditions (pH > 7)—Ce^4+^ is reduced to Ce^3+^, enhancing SOD-like activity and scavenging excess ROS produced by infiltrating neutrophils. This reduces oxidative stress, dampens prolonged inflammation, and promotes tissue repair [30,31,32]. Conversely, in acute wounds with acidic microenvironments (pH < 7), Ce^4+^ predominates, conferring pro-oxidant properties that inhibit bacterial growth and biofilm formation [33].

Thus, the therapeutic efficacy of cerium oxide nanoparticles is not absolute but contingent upon the precise control of their oxidation state during synthesis and careful consideration of the pathophysiological context of the wound, including pH, inflammatory phase, and microbial status, when designing nanoceria–based wound therapeutics.

In addition, nanoceria exhibit anti-inflammatory effects, which may be mediated through several interconnected mechanisms. According to multiple studies, nanoceria can modulate signaling pathways that drive the inflammatory response, for instance, by inhibiting the activity of Janus kinases (JAK) and mitogen-activated protein kinases (MAPKs) [34,35], and by regulating the expression of pro-inflammatory cytokine genes via the nuclear factor kappa B (NF-κB) transcription factor [36]. Consequently, nanoceria can suppress the production of key pro-inflammatory mediators such as interleukins (ILs) and tumor necrosis factor-alpha (TNF-α) [37,38].

Furthermore, the attenuation of the inflammatory response is also achieved by reducing the recruitment of phagocytic cells to the site of inflammation and by promoting their polarization toward an anti-inflammatory phenotype [39].

CeO_2_ nanoparticles also exert proliferative effects and stimulate angiogenesis while simultaneously inhibiting pathological neovascularization [40]. Multiple mechanisms govern angiogenesis. The previously described antioxidant and anti-inflammatory properties of nanoceria themselves contribute to a microenvironment conducive to the formation of new blood vessels. Additionally, the tissue oxygen concentration is a critical regulator of angiogenesis. Hypoxia activates hypoxia-inducible factor (HIF), which, in turn, upregulates vascular endothelial growth factor (VEGF), a master regulator of angiogenesis. Cerium oxide nanoparticles can modulate local oxygen availability, thereby enhancing VEGF gene expression and promoting neoangiogenesis [29,41,42]. However, once again, these pro-angiogenic properties appear to be primarily associated with the Ce^4+^ phase.

Currently, there is a substantial body of in vitro evidence demonstrating that nanoceria can enhance the maturation, migration, and proliferation of cells critical to wound regeneration (keratinocytes and fibroblasts) [43,44,45]. But at the same time, it is worth noting that conflicting data exist, with some in vivo studies reporting the opposite effect [46], which may be attributed to excessive and persistent immunosuppression induced by the nanoparticles.

Thus, it should be emphasized that the nanoceria efficacy is influenced by a wide range of factors, including the quality of the synthesized nanoparticles, their surface modifications, and the specific conditions of their application. Numerous in vitro studies have confirmed the diverse beneficial effects of nanoceria in wound regeneration [47]. However, data from in vivo studies, particularly those demonstrating therapeutic potential in living systems, are of exceptional interest and translational value, as they reflect the feasibility of clinical implementation [48,49,50]. Under physiologically relevant in vivo conditions, inflammatory and regenerative processes unfold dynamically and are accompanied by continuous shifts in key physicochemical parameters (pH, redox potential, cytokine levels, and microbial load). These changes can significantly alter the activity of both the cerium oxide nanoparticles and any auxiliary or modifying components within the final drug formulation, thereby necessitating careful consideration during the therapeutic design and evaluation.

### 2.2. Medical Devices and Pharmaceutical Formulations Based on Cerium Dioxide Nanoparticles

In the context of the biomedical applications of cerium oxide, primary attention should be given to the synthesis methods and physicochemical properties of the resulting nanoparticles, as well as to the excipients incorporated into the final pharmaceutical formulation (Table 1).

One of the most critical factors in harnessing the full potential of cerium dioxide (CeO_2_) lies in ensuring nanoscale dimensions during synthesis. This requirement stems from the ability of nanoparticles to traverse biological membranes, thereby enabling their biological effects. Particles exceeding 100 nm in size are generally discouraged because of their markedly reduced efficacy in interacting with cellular structures, including bacterial cells. Particles exceeding 100 nm in size are generally discouraged because of their markedly reduced efficacy in interacting with cellular structures, including bacterial cells [51,52]. Although a few studies have reported successful applications of larger particles [53,54], the prevailing consensus among researchers favors the use of nanoceria in the 3–30 nm range [55,56,57,58]. Within this size window, the material consistently exhibits robust antibacterial and antioxidant activities, which is attributed to its efficient penetration and interaction with both cellular and intracellular components [59,60,61,62].

In addition to particle size, the zeta potential (ζ-potential) significantly influences interactions with cellular structures through electrostatic forces. Negatively charged nanoparticles tend to be repelled by the intact, negatively charged cell membrane but may selectively bind to damaged cells exhibiting a positive surface charge. However, this parameter has not been consistently reported or considered in all studies. A similar mechanism can be applied to bacterial cell interactions. The zeta potential of a substance can be affected by various factors, such as the production method, electrolyte concentration, and pH. This has been demonstrated in a study by Patil et al. (2007), which found that the zeta potential influences the adsorption of cerium oxide nanoparticles on cells and their uptake [63]. Specifically, larger zeta potentials result in stronger binding, while negative zeta potentials do not seem to have any effect [63,64].

Consequently, comprehensive physicochemical characterization of the synthesized nanoparticles, including their size, size distribution, and zeta potential, is essential for a reliable preliminary assessment of their potential biological activity.

Currently, the following main types of synthesized nanocerium can be distinguished: spherical, cubic, rod-like, octahedral, rhombohedral, and spiked. Among these, the latter type has been shown to form nanobridges that can regenerate more efficiently than other forms [65]. The ratio of the particle’s surface area to its size, as well as the degree of cerium oxidation on the particle’s surface, have the most significant impact [66]. For instance, smaller spherical and octahedral particles that are close to a spherical shape exhibit better permeability through cell membranes compared to larger particles, due to their ability to penetrate cells through a non-volatilizable absorption pathway and maintain an equilibrium concentration of cerium in its III valence state both inside and outside the cell [67]. At the same time, rod-shaped nanoparticles exhibit better interaction with cellular surfaces, while maintaining the maximum amount of cerium present in its III oxidation state on the surface. However, it was the cubic structure that exhibited the greatest antioxidant activity, which the researchers attributed to the exposed surface area of the crystal, the predominance of Ce^4+^ ions on the surface, and the presence of Ce^3+^ ions in the interior of the nanoparticles [67].

The synthesis method has a significant influence on the physicochemical parameters of nanoparticles. The most commonly used method for preparing nanoceria in the reviewed studies was the hydrothermal method, which was used in six instances. Other methods, such as deposition, oxidation, and conventional wet-chemical synthesis, were also used, each occurring three times. Green synthesis was used in two studies, while the reverse micelle and modified Stöber methods were employed once [68,69]. One study employed a commercially available nanoparticle sample, and the synthesis method was not specified in the remaining cases. It is worth noting that the largest particle sizes were reported for nanoparticles synthesized by the hydrothermal method [70,71] and the oxidation methods [72], whereas the smallest particles were obtained via deposition [73,74].

In the designed medical products, nanoparticle concentrations ranged from very low levels (100 ng [75] and 0.0000156 wt% [73]) to relatively high loadings (1–5 wt% [68,69,76,77,78]). These medical aids typically incorporate cerium oxide nanoparticles either as active ingredients or as components within carrier matrices, often in combination with other excipients [68,74,79,80,81,82,83].

**Table 1:** Characteristics of cerium dioxide nanoparticles and nanoparticle-based formulations.

# No.	Characteristics of the Obtained CeO_2_ NP Formulation	Synthesis Method	NP Size (nm)	Hydrodynamic Radius (nm)	Zeta Potential (mV) ^1^	CeO_2_ NP Concentration	Excipients	Auxiliary Active Ingredients	References
	Solutions								
1	Solution	Simple liquid-phase chemistry method [55]	28.5± 0.8	3–5	ND	100 ng	PBS	miR146a	[75]
2	Solution	Simple liquid-phase chemistry method [55]	3–5	15–20	ND	ND	PBS	miR146a	[56]
3	Porous nanospheres in solution	Wet chemical method	100–200	143.4 ± 4.3	ND	ND	Distilled water	Copper	[83]
	**Suspensions**								
4	Colloidal dispersion	Deposition method	5.87 ± 1.27	ND	ND	0.0000156 wt% (1 µM)	Chitosan	Silver (5 or 7%)	[73]
5	Aqueous suspension	Hydrothermal method	104.3 ± 13.1	ND	8.8 ± 1.1 mV—with silicon and −10.0 ± 1.3 mV—hollow	10 mg mL^−1^	No	L-arginine, silicon dioxide	[70]
6	Suspension	qa	90 ± 6.4—hollow NP	ND	ND	1 mg mL^−1^	linker (N-Hydroxysuccinimide (NHS)-ester) i-motif DNA, MMP-cleavable stealth peptide	Graphene, arginine	[84]
	**Hydrogels**								
7	Hydrogel	Oxidation method	750–800	ND	ND	0.014 wt%. and 0.056 wt%. (0.1 and 0.4 mm, respectively)	Acrylamide, AMPS, MBA	Curcumin	[72]
8	Hydrogels	Oxidation method [58]	3–5	ND	ND	ND	Dextran, FITC, SBMA or CBMA, HEMA	miRNA146a	[57]
9	Hydrogel	ND	ND	ND	ND	ND	ND	ND	[85]
10	Hydrogel	ND	ND	ND	ND	ND	ND	ND	[86]
11	Hydrogel	Hydrothermal synthesis [87]	Rods: 9.6 ± 1.2 × (50–200)	ND	ND	PEI/PVP@CeO_2_ 0.5 wt%.	PEI, PVP, F127/F127-CHO	No	[88]
12	Hydrogel	ND	ND	ND	ND	1%	ND	ND	[77]
13	Gel	Hydrothermal method	400–450	ND	ND	ND	PHEM, Chitosan	No	[71]
14	Hydrogel	ND	ND	ND	ND	500 µg/mL	Gellan gum, gelatin	Flurbiprofen	[79]
15	Hydrogel	Green synthesis	18.8 ± 4.1	ND	ND	2 wt%.	Alginate	Curcumin	[68]
16	Hydrogel	Reverse micelle method [89]	3.3 [66]	18–30 [66]	ND	ND	ZIF-8, GelMA	Doxorubicin	[80]
	**Designed Products**								
17	Lyophilized sponge	Hydrothermal method [89]	2.5–6.5	195 ± 3	22.4 mV	0.025 wt%. (250 µg/mL)	Gelatin, genipin, oleylamine coating (stabilized)	No	[90]
18	Patches	ND	ND	ND	ND	1 wt%.	GelMA	No	[78]
20	Chitosan Hydrogel Membrane	Green synthesis	35–40	ND	ND	1% and 5% of Chitosan wt	Chitosan, glycerol	No	[69]
21	Sprayable Hydrogel Dressing	Purchased from US Research Nanomaterials	10–30	ND	ND	0.01 wt%. (100 µg/mL)	GelMA- dopamine	Antimicrobial peptides	[81]
22	Wound Dressing	Deposition method [91]	2–3	ND	−18.6 ± 2.59 mV	ND	PArg, DS, citric acid (stabilized)	Pirfenidone	[74]
	**Other**								
23	ND	Oxidation method	20 (190–CNP-miR146a)	20 (190–CNP-miR146a)	27 mV (−18mV–CNP-miR146a)	ND	Nanosilk	miR-146a	[82]
24	ND	Deposition method	45 (CS-ZnO/CeO_2_)	ND	ND	ND	Chitosan	ZnO	[92]

Note: ^1^ Reported for colloidal dispersions (sols). AMPS—acrylamido-2-methylpropane sulfonic acid, CBMA—3-[[2-(Methacryloyloxy)ethyl] dimethylammonio] propionate, CS— chitosan, DS—dextran sulfate sodium salt, F127—Pluronic F127, FITC—Fluorescein isothiocyanate, GelMA—gelatin-methacryloyl, HEMA—2-hydroxyethyl methacrylate, MBA—N′ methylene bisacrylamide, miR146a—MicroRNA 146a, MMP—matrix metalloproteinase, ND—Not Disclosed, NP—nanoparticles, PArg—poly-L-arginine hydrochloride, PBS—phosphate-buffered saline, PEI—polyethyleneimine, PHEM—Poly(hydroxyethylmethacrylate), PVA—polyvinyl alcohol, PVP—polyvinyl pyrrolidone, SBMA—[2-(methacryloloxy)ethyl]dimethyl-(3-sulfopropyl) ammonium hydroxide, ZIF-8—Zeolitic imidazolate framework-8, ZnO—zinc oxide, wt—weight, wt%—mass percent. The primary classes of auxiliary components used to stabilize or functionalize cerium oxide nanoparticles include chitosan [69,71,73,92], polyethyleneimine (PEI) and polyvinylpyrrolidone (PVP) [88], alginate [68], gelatin, and genipin [90]. Chitosan was the most widely adopted carrier matrix, applied in four studies [69,71,74,84], followed by gelatin (also in four studies: [79,80,81,90]).

Chitosan is one of the most widely used compounds for nanoparticle stabilization [69,71,73,92]. It offers high biocompatibility, biodegradability, low toxicity, favorable gas permeability, and intrinsic antibacterial activity, primarily mediated by electrostatic interactions between its positively charged amino groups [93,94] and the negatively charged bacterial cell membranes [95,96]. However, chitosan is inherently heterogeneous in molecular weight and degree of deacetylation, which limits its reproducibility and scalability in commercial biomedical applications because of the batch-to-batch variability in composite formulations [97,98].

The final pharmaceutical form also critically influences performance: most formulations are delivered either as dense dressings or suspensions [71,73,74,99]. Dense dressings require physical fixation and may cause re-traumatization upon removal, whereas suspensions lack sufficient viscosity and cohesion, necessitating additional measures to ensure adhesion to the wound site. Thus, a fully biodegradable wound dressing is an optimal solution to these challenges.

Gelatin is another commonly used matrix, often employed alongside chitosan [78,79,90]. However, in its native form, gelatin lacks mechanical strength (which typically restricts its use in hydrogels) and may exhibit insufficient porosity to support optimal cell adhesion, proliferation, and gas exchange, limiting its standalone biomedical utility [100,101]. These drawbacks can be overcome through fabrication techniques such as electrospinning, casting, or compression molding [102,103,104], and chemical crosslinking or blending with reinforcing agents (chitosan, polycaprolactone, or methacrylic anhydride) [100,105,106]. Such modifications preserve gelatin biocompatibility while endowing the resulting medical devices with enhanced absorptive capacity [107] and structural integrity [108].

PVA is a hydrophilic polymer that exhibits excellent film and gel-forming properties due to its physical or chemical crosslinking [109]. It is also biodegradable and non-toxic, which is extremely important for biomedical applications [110]. Additionally, compared to natural polymers, polyvinyl alcohol (PVA) has better physicochemical properties, such as high tensile strength and high Young’s modulus [111].

A modern and promising strategy involves the use of microRNAs (miRNAs), which function not only as immunomodulatory matrices but also exert intrinsic anti-inflammatory effects [56,57,75]. miRNAs enable targeted intracellular delivery of nanoparticles, thereby enhancing both anti-inflammatory and regenerative outcomes, primarily through modulation of the NF-κB signaling pathway [112,113,114,115].

Thus, excipients serve a dual purpose: first, they allow fine-tuning of the physicochemical properties of the final formulation and can significantly enhance the therapeutic potential of cerium-based nanoparticles [25]; second, many of these excipients possess their own inherent antibacterial or regenerative activities [116,117].

Moreover, in the analyzed studies, cerium oxide nanoparticles were frequently combined with additional active ingredients, including silver, antimicrobial peptides, doxorubicin, miR-146a, and curcumin [56,57,58,75]. However, considering the potential interactions among these components is crucial, as they may act synergistically or antagonistically, thereby altering or even diminishing the overall efficacy of the formulation. This underscores the necessity for systematic studies to evaluate the individual and combined biological effects of each constituent in multicomponent nanotherapeutics.

Another equally critical consideration is the final dosage form of the therapeutic product. In the studies reviewed, formulations were predominantly designed for transdermal or topical applications (hydrogels, wound dressings, patches, and sponges). Subcutaneous injection of a nanoceria-based formulation was reported in one study [88], whereas another employed the subcutaneous implantation of a cerium oxide-coated plate [77]. Aqueous solutions have also been used in several studies; however, their application is less suitable because of their poor retention at the wound site and lack of mechanical protection. In contrast, hydrogels and designed devices (dressings or patches) offer ease of application, better adherence, and simplified wound care, making them particularly well suited for localized wound therapy.

Therefore, the rational design of an optimal cerium dioxide nanoparticle-based formulation capable of supporting high-quality tissue regeneration requires a holistic approach that integrates multiple technological factors: synthesis method, stabilization strategy, nanoparticle physicochemical properties (size, zeta potential), nanoparticle concentration, final composition, dosage form, and the potential inclusion of additional active ingredients. The full therapeutic potential of nanoceria can only be reliably harnessed in clinical wound management through such a comprehensive framework.

### 2.3. Evaluation of the Efficacy of Cerium Dioxide Nanoparticle-Based Medical Devices in Wound Regeneration

As previously described, medical devices incorporating cerium dioxide nanoparticles have demonstrated promising outcomes in wound regeneration owing to their multifaceted therapeutic mechanisms. The efficacy of these nanomaterials must be critically assessed in the context of wound healing performance. Key outcome measures include the wound closure rate, tissue regeneration quality, wound infection incidence, and antibacterial potency of the nanoparticles. Therefore, the methodological rigor of a study, particularly its experimental design, is a crucial indicator of the reliability and translational relevance of its findings.

Table 2 summarizes the in vivo wound regeneration protocols employed in animal models across the reviewed studies, including wound characteristics, application methods of the cerium-based formulations, and healing assessment frequency.

Among the analyzed studies, five employed Sprague Dawley (SD) rats [68,71,75,81,84], seven used Wistar rats [73,77,78,84,85,91], two utilized BALB/c mice [70,72]; one used db/db mice [57]; and one study was conducted using a porcine model [57]. Rodents are commonly selected because of their relatively low cost and ease of handling, whereas porcine models, although being physiologically and genetically closer to humans, particularly in terms of skin architecture and wound healing mechanisms, are far more expensive to maintain. Consequently, despite its limited use, the porcine model holds high translational value.

**Table 2:** Methods for assessing wound regeneration in in vivo studies.

# No.	Focus of the Research	Number of Subjects	Wound Type	Wound Manipulation	Drug Administration Method	Control Groups (Drug-Free)	Control Groups (Versus Comparator)	Frequency of Control	Research Methods	References
Solutions										
1	Female ICR mice	ND	Surgical wound (d = 5 mm)	Treatment 12 h after injury, irradiation with white light	Transdermal	PBS	CNP-miR146a (100 ng)	0, 3, 7, 10, and 14	Wound closure assessment, tissue histology (Masson’s Trichrome), analyzing the numbers of CD31-positive and CD45-positive cells	[75]
2	BKS.Cg-Dock7m+/+Leprdb/J, strain No. 000642	ND	8 mm surgical wound biopsy punch	Topical application of active substance, then dressed with a Tegaderm (3M), which was subsequently removed on post-operative day 2	Transdermal	PBS (non-diabetics)	Diabetics: lenti-miRGFP (Control miR), lenti-miR146a, CNP-miR146a	every other day until wounds were fully closed	Wound closure assessment, tissue histology—immunohistochemistry	[56]
3	Male SD rats	ND	10 mm surgical wound biopsy punch	**a 10 μL suspension of *E. coli* (1.0 × 106 CFU/mL) was evenly applied to the wound surface**. Then, the wounds were treated with 200 μL of PBS or 200 μL of PBS containing different concentrations of Cu^2+^, HMCe, and Cu-HMCe solutions	Transdermal	PBS	Cu-HMCe, HMCe, Cu	Daily before 14th day	Wound closure assessment, histology (H&E staining, Masson staining—14th day; immunohistochemical analysis TNF-α, IL-6, CD31—3 and 10 days); neovascularization ability—α-SMA and CD31	[83]
**Suspensions**										
4	albino mice	12	4 mm surgical wound biopsy punch	Wound treatment every 24 h with removal of wound crusts	Transdermal	Off-dose	Ag–CeO–Chitosan (5 and 7%), Ag–Chitosan	0, 30, 60, 90 days after the wound was inflicted	Wound closure assessment, collagen density assessment (Masson’s), wound microbial load assessment	[73]
5	Female BALB/c mice	ND	Surgical wound	Applying a drop to the wound and pressing the wound for 30 s	Transdermal	PBS	hCeO_2_ NPs, AhCeO_2_ NPS, AhCeO_2_ NPS + simulated sunlight irradiation	0, 2, 4, 6, 8, 10 days after surgery	Wound closure assessment, histology (H & E)	[70]
6	Female ICR mice	ND	Surgical wound 0.5 cm × 0.5 cm	Application of 50 μL of NC CG or ACG suspension with a concentration of 1 mg/mL (equivalent to cerium) in PBS after 12 h	Transdermal	PBS	CG NCs, ACG NCs	12, 24 h, 2nd–14th day, every other day	Wound closure assessment, tissue histology (H & E), collagen content assessment—hydroxyproline assay	[84]
**Hydrogels**										
7	Male Wistar rats	30	Surgical wound (d = 10 mm)	Application of round hydrogel scaffolds followed by covering with a transparent gauze dressing (Medicare B.P.) and a sterile adhesive dressing (Medicare)	Transdermal	No	Medicare cotton wool, A; AC; AC’; ACC	0, 7, 3 and 14 days	Wound closure assessment, collagen density assessment (Masson’s), stage evaluation (H & E)	[72]
8	12-week Db/Db female mice	10	Surgical wound l = 8 mm	Single application of gel to the wound	Injection therapy	No	hydrogel, CNP-miR146a	In 1 day until 20th day	Wound closure assessment, biomechanical skin testing, gene expression	[57]
9	Male Wistar rats	30	Surgical wound **non-sterile**	Treatment after injury and on days of intermediate control, covering with a sterile plaster	Transdermal	Off-dose	Hydrogel	0, 1, 2, 3, 4, 5, and 7; 14—euthanasia day	Wound closure assessment, wound histology (Masson’s)	[85]
10	Wistar rats	ND	3rd-degree burn wound.	Application to a burn wound	Transdermal	Off-dose	Levomecol, intact gel, CNP-doped gel	5, 25	Wound closure assessment	[86]
11	Female mice	ND	7 mm surgical wound biopsy punch	Treatment of the wound with the active substance	Injection therapy	Off-dose	3M Tegaderm, FVEC-0, FVEC-1 (0.5%)	0, 3, 8 and 14 days	Wound closure assessment, histology (H&E)	[88]
12	Male Wistar rats	20	Surgical wound line-like, l = 45 mm	The wound was sutured and the composition was applied once daily. The stitches were removed on the 10th day.	Transdermal	No	Hydrogel, hydrogel c CNP	Daily until 21st day	Wound closure assessment, mechanical characteristics of skin	[77]
13	Female SD rats	8	Surgical wound (d = 1 cm)	Single application to the wound	Transdermal	Off-dose	PHEM-CS gel, cerium-doped gel, PHEM-CS-CNP	2, 6, 10, and 14 days	Wound closure assessment, tissue histology (H&E)	[71]
14	Rats	ND	ND	Application of the composition to the wound	Transdermal	Off-dose	Paraffin and material treated group (GG/Ge and GG/Ge/NC FLU)	0, 3, 7, 11 and 14	Wound closure assessment, tissue histology	[79]
15	Male SD rats	ND	10 mm surgical wound biopsy punch	Application of the composition to the wound	Transdermal	Off-dose	Pure alginate hydrogel, Alg/CeO NPs 3%, Alg/CeO NPs 5%, Alg/CeO NPs 7%	0, 14	Wound closure assessment, tissue histology (H&E)	[68]
16	Male SD rats	3	8 mm surgical wound biopsy punch	Application of the hydrogel to the wound	Transdermal	PBS	GelMA, and ZC@GelMA	0, 3, 6, 9, 12	Wound closure assessment, histology (H&E, Masson)	[80]
**Designed products**										
17	Female Wistar rats	24	Surgical wound	Application of a sponge under the bandage	Transdermal	Off-dose	Gelatin with cerium oxide NP, gelatin	0, 4, 8 and 12-th days after surgery	Wound closure assessment, collagen density assessment (Masson’s), lymphocytic infiltration assessment (H & E)	[90]
18	Diabetic rats	ND	ND	Application of the composition to the wound	Transdermal	PBS	Standard dressing (Puracol Plus-Ag+, Medline), GelMA gel, GelMA − CONP-1 patches	0, 3, 7, 10, 30 + daily assessment	Wound closure assessment, histology—daily examination of the wound; tissue histology (H & E)	[78]
19	Male Swiss albino mice	12	Surgical wound (d = 2 cm)	Daily treatment of the wound with the active substance	Transdermal	No	Membrane, 1% CNP membrane, 5% CNP membrane	4, 7, 11, 15	Wound closure assessment	[69]
20	NS	ND	8 mm surgical wound biopsy punch	***S. aureus*-induced wound infection**	Transdermal	No	gel, gel + AMP, gel + CNP, gel + AMP + CNP	0, 3, 7, 14	Wound closure assessment	[81]
21	Male SD rats	25	6 mm surgical wound biopsy punch	Application of dressings with various substances	Transdermal	Off-dose	PLA; PFD NCs + PLA; CeO_2_ NCs + PLA; PFD/CeO_2_ NCs + PLA	Every 2 days before 14th	Wound closure assessment, tissue histology	[74]
**Other**										
22	BKS.Cg-Dock7m+/+Leprdb/J, strain No. 000642	12–15	8 mm surgical wound biopsy punch	Topical application of active substance, then dressed with a Tegaderm (3M), which was subsequently removed on post-operative day 2	Transdermal	PBS	NS, NS-CNP-miR146a	10, 12, 14, 16	Wound closure assessment, tissue histology (Masson’s Trichrome), gene expression	[82]
23	Male Wister albino rats	9	Biopsy punch	Wound covering	Transdermal	Off-dose	CS-ZnO hybrid composite, CS-ZnO/CeO_2_ hybrid nanocomposite	Daily until 21st day	Wound closure assessment, mechanical skin characteristics	[92]

Note: A—hydrogel, AC—CNP-entrapped A hydrogel, ACC—curcumin-entrapped CNP-loaded hydrogel, ACG NCs—ceria NP-detachable graphene nanocomposites, AC’—curcumin-loaded void A hydrogel, AhCeO_2_ NPs—l-arginine inside CeO_2_ NPs, AMP—antimicrobic peptide, CD31—Platelet endothelial cell adhesion molecule-31, CFU—colony-forming unit, CG NCs—ceria-graphene nanocomposites, CNP—cerium nanoparticles, CS- chitosan, Cu-HMCe—copper-doped hollow mesoporecerium oxide, FVEC-0 or FVEC-1—PEI/PVP@CeO_2_ 0 wt% or 0.5 wt, GelMA—gelatin methacryloyl, GG/Ge/NC@FLU—nanoceria and flurbiprofen-loaded hydrogel, H&E—Hematoxylin and eosin stain, hCeO_2_—hollow CeO_2_ NPs, ICR—Institute of Cancer Research, miR146a—MicroRNA 146a, IL-6—Interleukin-6, No—there was no comparison group, ND—Not Disclosed, NS—nanosilk, PBS—phosphate-buffered saline, PFD—pirfenidone, PHEM—Poly(hydroxyethylmethacrylate), PHEM-CS—poly(2-hydroxyethylmethacrylate)-chitosan, PLA—polylactic acid, SD—Sprague-Dawley, SMA—smooth muscle actin, TNF—Tumor Necrosis Factor, ZC—zeolitic imidazolate framework-8@ceric oxide nanoparticle.

The method of wound induction, which should closely mimic clinically relevant injury scenarios is equally critical. The reviewed studies employed the following wound models:

- seven surgical (line-like incision) wounds [57,69,71,72,73,77,90];
- twelve excisional (full-thickness) wounds simulated traumatic injury [56,58,68,72,73,74,80,81,83,88,92];
- one burn wounds [86];
- three models of chronic wounds [56,78,82].

Diabetic wounds represent the most feasible chronic wound model in animals because venous ulcers or pressure sores are difficult to reproduce reliably in preclinical settings. Notably, only three studies created acute wounds under strictly aseptic conditions, better reflecting clean traumatic injuries [81,83,85]. Furthermore, one study disrupted the natural healing process by daily crust removal and tissue damage, which was a significant methodological limitation that compromised comparability with other studies [77].

In five studies [56,58,72,85,90], the wound protocol followed a standardized clinical-like procedure: the wound site was disinfected, the test formulation was applied directly to the wound, and a dressing or adhesive patch was placed on top of the wound. While this fact mirrors routine first-aid care for minor injuries, it may not reflect real-world scenarios such as battlefield or disaster-related trauma, where wounds are often contaminated. Thus, a sterile dressing was applied in one study, but the wound was created under non-sterile conditions [85], offering a more realistic compromise. Wound induction under sterile conditions enables a clearer assessment of the intrinsic tissue healing quality by minimizing confounding variables, such as microbial contamination. However, because most real-world traumatic injuries involve some degree of tissue contamination or infection risk, studies that incorporate this factor offer greater clinical relevance. Moreover, contaminated or infected wound models allow for a more comprehensive evaluation of the dual functionality of cerium oxide nanoparticles, namely, their concurrent antibacterial and regenerative effects, which are mediated by the redox-dependent interplay between Ce^3+^ and Ce^4+^ states.

Remarkably, cerium-based formulations were applied in twenty studies without any secondary dressing [57,58,72,86,118]. The observed efficacy in these cases suggests the potential for “dressing-free” therapeutic use, which could (1) minimize iatrogenic tissue trauma during dressing changes while maintaining a protective barrier, and (2) reduce overall wound care costs.

Two studies deliberately inoculated wounds with bacterial suspensions [81,83], which is a non-physiological but useful approach for rigorously assessing antimicrobial and immunomodulatory potential. Intriguingly, despite robust antibacterial activity and stimulation of neoangiogenesis, hollow mesoporecerium oxide also significantly attenuated the inflammatory response [92], supporting the hypothesis that cerium redox activity (Ce^3+^/Ce^4+^ switching) is dynamically regulated by the local wound microenvironment.

Finally, Luo et al. [71] surgically closed wounds with sutures, modeling a postoperative scenario. This design uniquely enabled the evaluation of interactions between the cerium composite and suture materials, which is relevant for implantable or post-surgical applications.

Wound induction under sterile conditions enables a clearer assessment of the intrinsic tissue healing quality by minimizing confounding variables, such as microbial contamination. However, because most real-world traumatic injuries involve some degree of tissue contamination or infection risk, studies that incorporate this factor offer greater clinical relevance. Moreover, contaminated or infected wound models allow for a more comprehensive evaluation of the dual functionality of cerium oxide nanoparticles, namely, their concurrent antibacterial and regenerative effects, which are mediated by the redox-dependent interplay between Ce^3+^ and Ce^4+^ states.

The majority of the reviewed studies (18 out of 24), included a negative control group (treatment-free animals). However, comparative groups typically comprised alternative experimental formulations rather than clinically established benchmarks. Only four studies employed commercially available wound dressings as active comparators, which is critical for assessing the translational and industrial potential of nanoceria-based products. Such comparisons are particularly valuable for evaluating whether novel nanoceria formulations offer either superior clinical performance or comparable efficacy at lower production costs than existing market solutions.

Specifically, head-to-head evaluations were conducted using Levometil ointment [86], Medicare^®^ commercial cotton wool [72], Puracol^®^ and Medline^®^ dressings [78], and 3M Tegaderm™ [88]. Cerium oxide-containing composites demonstrated superior healing outcomes compared to Unguentum chloramphenicol, Medicare^®^ cotton wool, and 3M Tegaderm™. However, the silver-containing commercial dressing Puracol^®^ outperformed the experimental cerium-based formulation in one study, highlighting the continued competitiveness of the established antimicrobial dressings in specific contexts.

#### 2.3.1. Assessment of Wound Healing Rate

The assessment of wound healing may rely on both subjective and objective parameters and can focus on either the rate or quality of tissue repair. Measurement of wound closure area is one of the most accessible and widely used methods in in vivo studies. However, this approach has limitations: it is inherently prone to observer bias and becomes technically challenging when wounds exhibit irregular or complex geometries. Digital image analysis software can be employed for automated wound area quantification and the longitudinal comparison of standardized photographs to enhance the research objectivity.

Histological analysis provides a more detailed assessment, enabling the evaluation of inflammatory infiltration, cellular composition (leukocytes, macrophages, and fibroblasts), and collagen fiber organization and orientation. Additionally, biomechanical testing, such as tensile strength measurements (“burst strength” or stress–strain testing) or ultrasound elastography, offers valuable insights into the functional integrity of healed tissue by quantifying parameters including tensile strength, elasticity, and Young’s modulus. Among these approaches, the wound closure rate is the most obvious way to evaluate the efficacy of a wound care product. Table 3 summarizes the outcomes of applying various cerium-based medical formulations to accelerate wound healing.

The fastest healing time reported was 6 ± 2 days; however, this result must be interpreted with caution, as the wound model involved a linear incision followed by surgical suturing, which inherently accelerates closure compared to non-sutured excisional wounds [77]. Obviously, sutured surgical wounds consistently heal more rapidly than punch-biopsy or full-thickness excisional models without closure [70,90]. In the case of punch-biopsy wounds, the shortest healing duration was 12 days [92]; however, the formulation tested in that study comprised a multi-component system (CS–ZnO/CeO_2_), precluding an isolated assessment of the regenerative contribution of cerium oxide nanoparticles.

In most studies, complete wound closure was achieved within approximately 14 days, regardless of the specific experimental formulation. While all cerium-containing formulations outperformed the negative controls (treatment-free or PBS-treated animals), superior outcomes were consistently observed with either multi-active formulations or the commercial silver-containing dressing Puracol Ag [78].

Scaffolds or carriers functionalized with nanoceria demonstrated statistically significant acceleration of wound healing compared to both negative controls (treatment-free or PBS) [80,81,84] and positive controls such as Levomecol [86] or 3M Tegaderm [88]. Nevertheless, their clinical relevance is best assessed through comparison with the established positive controls, as untreated wounds are rarely encountered in real-world clinical practice [119,120].

A key limitation across many studies is the inability to attribute the observed regenerative effects solely to cerium oxide nanoparticles because of the frequent use of multicomponent formulations [57,71,73,75,79,82,88] and the absence of parallel experimental arms testing cerium-only formulations under identical conditions. This confounding factor hinders definitive conclusions regarding the intrinsic regenerative potential of the nanoceria.

#### 2.3.2. Assessment of Regeneration Quality

In addition to the wound closure rate, the tissue restoration quality is a critical determinant of therapeutic success. Thus, poor-quality healing, characterized by the deposition of coarse disorganized type IV collagen fibers, can result in esthetically undesirable scarring and may impair local microcirculation and innervation [121].

Regeneration quality can be evaluated using both subjective and objective approaches. Subjective methods included visual inspection of the healed area and scar dimension measurement. However, none of the reviewed studies relied solely on such qualitative assessments. Instead, more rigorous and informative evaluations were employed, including histological analysis (Table 4), immunohistochemical and molecular-genetic assays (Table 5), quantitative collagen content determination (notably absent in most studies), and biomechanical testing compared to native skin properties (Table 6).

The classical inflammatory response in wound healing implies early neutrophil–macrophage infiltration. These immune cells not only defend against wound infection but also secrete a balanced repertoire of pro- and anti-inflammatory mediators that govern subsequent regenerative processes. Histological analyses consistently revealed a neutrophil–lymphocyte infiltrate persisting for at least the first week post-injury, followed by a gradual transition toward fibroblast-dominated granulation tissue engaged in collagen synthesis [74,79,85]. However, in several studies, inflammation persisted beyond day 14, extending up to day 24, indicating dysregulated immune resolution and an elevated risk of chronic inflammation [70,72,84,122]. Moreover, one study interpreted the presence of macrophages on day 24 as a sign of prolonged composite matrix degradation and a potential foreign-body immune response to scaffold components [84].

Histology demonstrated increased collagen deposition and greater hair follicle regeneration in the cerium-treated groups compared to the controls. Nevertheless, the quality of the newly formed epidermis was generally inferior to that observed with multi-active formulations, as evidenced by coarser collagen structure and less effective microbial suppression [71,74,85]. These findings suggest that cerium oxide nanoparticles possess intrinsic regenerative potential, although submaximal, which could be enhanced through strategic formulation or scaffold modification [18,29,120,123,124]. Complete re-epithelialization, being a key determinant in minimizing scar formation, was achieved in only 6 of the reviewed studies [71,74,78,85,88,92]. In the remaining cases, the epithelial coverage was partial, indicating a high likelihood of scar development at the wound site. A consistently positive effect of nanoceria was observed on neoangiogenesis: nearly all studies reported significantly enhanced angiogenesis relative to both negative and positive control groups, including those employing multicomponent therapeutics.

Gene expression profiling and cytokine activity assays provide deeper mechanistic insights into the wound microenvironment at the molecular level (Table 5).

The pro-angiogenic effect of cerium oxide nanoparticles was further corroborated by immunohistochemical analyses of vascular endothelial growth factor receptor (VEGFR) and CD31 (an endothelial cell marker), which consistently demonstrated significantly elevated expression levels in treated groups [125].

In most studies, nanoceria-containing formulations were associated with a marked reduction in inflammatory markers. Specifically, pro-inflammatory cytokines, including IL-6, IL-8, MCP-1, CD45, and CXCL2, were significantly downregulated following treatment [108]. Conversely, anti-inflammatory mediators such as TGF-β and IL-10 were upregulated [72,79,82], indicating a shift toward a regenerative, immunomodulatory microenvironment.

Collagen synthesis and tissue remodeling were further assessed by the expression of Col1a2, the gene encoding the α2 chain of type I collagen. A substantial increase in Col1a2 expression was observed across multiple studies, supporting robust extracellular matrix deposition and favorable wound-healing outcomes. Collectively, these molecular and histological findings confirm the dual beneficial role of cerium oxide nanoparticles in promoting neoangiogenesis and suppressing excessive inflammation [56,57,72,79,82].

In several studies, the biomechanical integrity was evaluated using tensile strength (“burst strength”) testing (Table 6). However, two reports omitted data on the mechanical properties of intact, uninjured skin, thereby lacking a critical reference for assessing the quality of the healed tissue. In studies that included this control, the healed tissue achieved 77–80% of the tensile strength of native skin, which is considered excellent in the context of dermal regeneration [79,90].

#### 2.3.3. Antibacterial Effect

Antimicrobial activity is an essential attribute of advanced wound dressings is, which helps prevent infection and supports a conducive environment for healing. Cerium oxide exhibits a limited intrinsic antibacterial activity under physiological conditions [126]. This limitation stems from several interrelated factors: (i) the Ce^3+^ oxidation state, which is associated with antibacterial activity, is thermodynamically less stable than Ce^4+^; (ii) infected wounds typically present a mildly alkaline microenvironment, which favors the formation of CeO_2_ (Ce^4+^-dominant) and suppresses the Ce^3+^ fraction [127]; and (iii) the concentration of cerium required to achieve significant bactericidal effects may approach cytotoxic thresholds for mammalian cells. Nevertheless, the Ce^3+^ state may contribute to mild bacteriostatic activity, as suggested by several studies [128,129,130].

Among the reviewed studies, only a few cases directly assessed the antibacterial efficacy of nanoceria. According to [81], cerium was combined with an antimicrobial peptide; however, the observed antibacterial effect was attributed solely to the peptide, with no synergistic enhancement conferred by cerium. The lack of additional antimicrobial activity was likely caused by the low cerium concentration used (100 µg/mL).

Similarly, cerium oxide alone was compared with a cerium-copper composite in [84]. After 24 h, nanoceria alone achieved only ~20% inhibition against *aureus* (Gram-positive bacteria) and ~30% inhibition against *Escherichia coli* (Gram-negative bacteria), which was significantly lower than that of the copper-containing formulation.

Other studies on antibacterial activity have shown a significant effect on Gram-negative bacteria such as Klebsiella pneumoniae [131], *P. aeruginosa* [132,133]. Gram-positive bacteria have a thicker cell wall, which reduces the effectiveness of CEO_2_ nanoparticles against them by inhibiting various pathogenic mechanisms. These mechanisms include intracellular induction of reactive oxygen species (ROS), direct damage to the bacterial cell wall, and interference with cellular respiration [61,134].

Depending on the shape of the cerium oxide nanoparticles, they may also have an antifungal effect against fungi such as *Aspergillus elegans*, *Aspergillus niger*, and *Trichophyton rubrum* [66].

Thus, these findings indicate that, at the concentrations and formulations typically employed in wound healing studies, cerium oxide nanoparticles exert, at best, a weak bacteriostatic effect that is insufficient to serve as a primary antimicrobial agent. However, given the limited number of dedicated antimicrobial evaluations and the context-dependent redox behavior of cerium, further systematic studies under standardized infection-relevant conditions are warranted to fully elucidate its potential role in infection control.

## 3. Discussion

A total of 25 experimental in vivo studies were analyzed, all of which employed nanoceria either as the primary active ingredient or as a component of a multifunctional formulation in medical devices designed to accelerate cutaneous regeneration. The rational design of such therapeutic products requires careful consideration of several critical factors starting from the earliest development stages.

The first key factor is the synthesis method, which directly determines the physicochemical properties of the resulting nanoparticles. Notably, the hydrothermal synthesis method was associated with the highest rates of wound closure and the best quality of regenerated epidermis across multiple studies, irrespective of the final particle size [71].

Another crucial aspect involves the choice of stabilizers and carrier matrices into which nanoceria are incorporated. The most frequently used matrices included chitosan, gelatin, and microRNAs [56,69,71,73,75,78,80,92]. These components were selected for their high biocompatibility and ability to preserve the functional properties of the nanoceria. Nevertheless, the stabilizer and matrix can significantly influence the healing outcomes through their physicochemical characteristics. Importantly, this interaction is not always adequately addressed in the literature. For instance, many commonly used stabilizers impart a negative surface charge to the nanoparticle formulations [55], which may hinder the biological activity of nanoceria, particularly its antibacterial effects, by reducing electrostatic attraction to negatively charged bacterial membranes or limiting cellular uptake.

The selection of the final dosage form is critical not only for user convenience but also for providing adequate mechanical protection of the wound site. Among the evaluated formulations, hydrogels [57,71,79], sponges [90], membranes [69], and liquid solutions [75] demonstrated favorable healing outcomes. However, hydrogels and structured devices, such as sponges, sprayable dressings, and films, are of particular interest because of their ease of storage, handling, and application in clinical or preclinical settings. In addition, in several studies, therapeutic efficacy was further enhanced by incorporating additional bioactive agents, including doxycycline, copper, and silver [73,80,83], which accelerated wound closure and improved the quality of regenerated tissue, as evidenced by increased collagen content, improved fiber organization, and enhanced cellular infiltration.

The majority of in vivo studies employed rodents, primarily mice and rats, as they represent the most accessible and cost-effective animal models. Healing was assessed through multiple complementary endpoints: wound closure rate, collagen deposition, inflammatory cell infiltration, pro-inflammatory cytokine levels, presence of keratinocytes and epithelial cells, extent of scar formation, and degree of neoangiogenesis in the newly formed tissue.

Collectively, these analyses demonstrated that cerium oxide nanoparticles consistently promote wound healing, primarily by accelerating the regenerative process. As previously reported by other investigators, this beneficial effect is likely attributable to the potent antioxidant activity of nanoceria, mediated through redox cycling between Ce^3+^ and Ce^4+^ states in the wound microenvironment coupled with their capacity to attenuate inflammatory responses [43,47]. These mechanisms collectively promote key regenerative processes, including keratinocyte migration and proliferation, collagen synthesis, tissue neovascularization, and reduced lymphocytic infiltration.

A particularly significant contribution of nanoceria to wound healing is their potent pro-angiogenic activity. Among all the tested formulations, nanoceria demonstrated the strongest stimulatory effect on neoangiogenesis, as evidenced by studies showing sustained enhancement of vascular regrowth via modulation of the ASK1–p38/JNK–NF-κB signaling axis [40]. This property enables the consideration of the potential of nanoceria for the treatment of chronic wounds, where restoration of the microvascular network is critical for achieving durable healing outcomes.

Comparative studies against commercially available wound dressings have generally demonstrated the superiority of experimental cerium-based formulations, highlighting their high therapeutic potential. However, this advantage was not universal. In one study, a cerium-containing formulation failed to outperform the silver-loaded commercial dressing Puracol Plus in terms of wound closure rate [70]. Moreover, combination strategies incorporating additional active agents, such as Ag, Cu, and miR146a, often yielded superior results compared to cerium oxide alone. For instance, a CeO_2_-copper composite achieved 90% wound closure, compared to 80% with cerium oxide monotherapy [49], indicating that synergistic formulations may be necessary to fully exploit the regenerative capacity of nanoceria.

In vivo studies have demonstrated the efficacy of nanoceria in promoting healing across diverse wound types, including burns, acute surgical wounds, chronic ulcers, and infected injuries. However, the current body of evidence remains limited in scope and depth. Further study of the regenerative potential of nanoceria requires comprehensive investigations to systematically evaluate how the composition of composite materials influences their biological activity.

Future studies should adhere to the following methodological principles to enhance the translational relevance and data reliability:

(1) Component-wise control groups must be included to isolate and quantify the individual contribution of each formulation constituent to wound healing;
(2) Species-specific limitations of rodent models, particularly the presence of a panniculus carnosus and dense hair follicle network, must be acknowledged and addressed using modified protocols to improve extrapolation to human wound healing;
(3) Full-thickness excisional (punch biopsy) wounds should be adopted as a standardized, reproducible model for assessing regenerative outcomes;
(4) Efficacy assessments should extend beyond sterile wound models to include wounds created under non-sterile conditions and those deliberately inoculated with pathogens to reflect real-world clinical scenarios;
(5) Chronic wound models, particularly those involving genetically or chemically induced diabetes mellitus, should be prioritized to evaluate the therapeutic potential of nanoceria in impaired healing contexts.

Implementation of these design criteria will generate robust and clinically meaningful data to support the rational development and eventual translation of nanoceria-based therapies into regenerative medicine and routine wound care practice.

## 4. Conclusions

Considering the presented evidence, cerium dioxide nanoparticles represent a promising therapeutic platform for wound healing applications. Their multifunctional biological activities, including antioxidant, anti-inflammatory, and pro-angiogenic properties, make them valuable candidates for the development of advanced regenerative medical devices. However, the realization of their full clinical potential hinges on the precise control of numerous interdependent factors. These include the synthesis method, which dictates core physicochemical characteristics such as particle size, crystallinity, and Ce^3+^/Ce^4+^ ratio; the choice of stabilizers and carrier matrices, which influence colloidal stability, biocompatibility, and bioactivity; and the final dosage form, whether hydrogel, sponge, membrane, or spray, which determines applicability, retention, and mechanical protection at the wound site.

Moreover, robust preclinical validation requires a tiered experimental approach that integrates in vitro cytocompatibility and mechanistic studies, ex vivo tissue models, and well-designed in vivo animal trials. Among these, in vivo studies are particularly critical, as they enable a holistic assessment of healing dynamics, including re-epithelialization, collagen remodeling, neovascularization, and immune modulation, under physiologically relevant conditions. Animal models, especially those mimicking chronic or infected wounds, provide indispensable insights to bridge the gap between benchtop innovation and clinical translation.

Nevertheless, the current literature remains fragmented, with limited standardization in experimental design, insufficient comparative data against established commercial dressings, and few studies isolating the specific contribution of nanoceria in multicomponent formulations. Consequently, while the therapeutic promise of cerium oxide nanoparticles is evident, a coordinated, systematic research effort, guided by rigorous methodological standards and clinically relevant endpoints, is essential to advance this technology toward regulatory approval and real-world implementation in wound care.

## Figures and Tables

**Table 3:** Comparison of experimental formulations by wound closure rate.

No.	Active Ingredient	Day	% Wound Closure	References
2	CeONPs	12	100%	[90]
3	CeONPs	10	88–100% (depending on NP form)	[70]
4	CeONPs	14	89%	[72]
5	CeONPs	14	79.1 ± 0.6%	[84]
6	CeONPs	14	94.7%	[85]
7	CeONPs	15	75%	[78]
		20	97%	
9	CeONPs	15	5%CeO—95%	[69]
			1%CeO—62%	
10	CeONPs	14	87.5%	[81]
11	CeONPs	14	90%	[74]
12	CeONPs	6 ± 2	100%	[77]
13	CeONPs	14	86.7%	[79]
14	CeONPs	14	80%	[83]
15	CeONPs	25	100%	[86]
Multicomponent active substances				
16	12GEL ZIF-8 CeO_2_-loaded GelMA	12	90%	[80]
17	PHEM-CS/CeONPs	14	98.5 ± 4.95%	[71]
18	CS-ZnO/CeO_2_	12	100%	[92]
19	0.5%PEI/PVP CeO_2_	14	100%	[88]
20	CNP-miR146a	14	100%	[56]
21	CNP-miR146a	14	97%	[82]
		16	100%	
22	CNP-miR146a	14	60%	[75]
23	CNP-miR146a	14	100%	[57]

Note: CeONP—cerium oxide nanoparticles, CNP—cerium nanoparticles, CS—chitosan, miR146a—MicroRNA 146a, PEI—polyethyleneimine, PHEM-CS—poly(2-hydroxyethylmethacrylate)-chitosan, PVP—polyvinyl pyrrolidone, ZIF-8—Zeolitic imidazolate framework-8, ZnO—zinc oxide.

**Table 4:** Histological features of healed wounds following treatment with nanoceria-based formulations.

# No.	Active Ingredient	Day	Exudation	Intact Epidermis	Collagen	Hair Follicle	Neoangiogenesis	References
1	CeONPs	14	ND	+	+ immature	+	+	[74]
2	CeONPs	14	ND	+	±	±	++	[57]
3	CeONPs	18	++	±	ND	ND	±	[72]
		24	−	+	ND	ND	++	
5	CeONPs	10	ND	±	+	ND	ND	[90]
6	CeONPs	14	ND	±	±	−	ND	[70]
7	CeONPs	14	ND	±	+	±	ND	[73]
8	CNP-miR146a	14	ND	±	ND	ND	++	[84]
9	CeONPs	3	+	±	+	ND	ND	[85]
		7	±	±	+	+	+	
		14	−	+	+ (immature)		++	
10	CeONPs	20	−	+	ND	+	+	[78]
11	0.5%PEI/PVP CeO_2_	14	−	+	ND	+	ND	[88]
12	CS-ZnO/CeO_2_	6	ND	ND	+	ND	ND	[92]
		12	ND	+	+	ND	ND	
13	PHEM-CS/CeONPs	14	ND	+	ND	+	ND	[71]
14	CeONPs	7	+	ND	ND	ND	+	[79]
		14	−	±	++	+	+	
15	CeONPs	14	ND	±	±	ND	ND	[83]

Note: Scoring: “−“ = absent; “±” = weak/discontinuous; “+” = present/continuous; “++” = abundant/continuous; CeONP—cerium oxide nanoparticles, CNP—cerium nanoparticles, CS—chitosan, miR146a—MicroRNA 146a, ND—Not Disclosed, PEI—polyethyleneimine, PHEM-CS—poly(2-hydroxyethylmethacrylate)-chitosan, PVP—polyvinyl pyrrolidone, ZnO—zinc oxide.

**Table 5:** Effect of cerium dioxide nanoparticles on cytokine and growth factor gene expression.

# No.	Active Ingredient	Il6	Il8	Il10	TGF-β	VEGFR	MCP-1	CD45	CD31	CXCL2	Col1a2	References
1	CeONPs	↑	ND	↑	↑↑	↑↑	↓	ND	ND	ND	ND	[72]
2	CNP-miR146a	ND	ND	ND	ND	ND	ND	↑	↑↑	ND	ND	[84]
3	CNP-miR146a	**↓↓**	ND	ND	ND	ND	ND	ND	ND	**↓↓**	↑↑	[57]
4	CNP-miR146a	**↓↓**	↓↓	ND	↑↑	ND	ND	↓	ND	ND	↑↑	[82]
5	CNP-miR146a	**↑↓**	ND	ND	ND	↑↑	ND	↓	↑	ND	ND	[56]
6	CeONPs	ND	ND	↑	↑	↑↑	ND	ND	ND	ND	ND	[79]
7	CeONPs	**↓**	ND	ND	**↓**	ND	ND	ND	↑	ND	ND	[83]

Note: ↓—decreased expression, ↑—increased expression, ↑↑—highly increased expression, **↓↓**—significant decreased expression, **↑↓**—no changes in expression, CeONP—cerium oxide nanoparticles, CNP—cerium nanoparticles, miR146a—MicroRNA 146a, ND—Not Disclosed.

**Table 6:** Physicomechanical properties of healed skin following treatment with nanoceria-based formulations.

# No.	Active Ingredient	Day	Tensile Strength of Skin, MPa		Young Modulus, MPa	% of the Norm	References
1	CeONPs	24	6.09 ± 0.23	7.85 ± 0.12	ND	77.6%	[90]
2	CNP-miR146a	14	ND	ND	8.5–13.7	ND	[84]
3	CNP-miR146a	14	2.5	4	22	55%	[57]
4	CNP-miR146a	18	ND	ND	99.67 ± 3.316	ND	[82]
5	CeONPs	12	4.18	5.109	ND	81,8%	[79]

Note: CeONPs—cerium oxide nanoparticles, CNP—cerium nanoparticles, miR146a—MicroRNA 146a, ND—Not Disclosed.

## Data Availability

The data supporting the findings of this study are available within the paper.

## Abbreviations

The following abbreviations are used in this manuscript:

AMR	Antimicrobial resistance
CeO_2_ NPs	Cerium oxide nanoparticles
PEI	Polyethyleneimine
PVA	Polyvinyl alcohol
PVP	Polyvinylpyrrolidone
ROS	Reactive Oxygen Species
VEGF	Vascular Endothelial Growth Factor
